# Hemorrhagic Stroke Revealing a Snake Bite: A Case Report

**DOI:** 10.7759/cureus.20935

**Published:** 2022-01-04

**Authors:** Keita Ansoumane Hawa, Jihane Mhaili, Badr Boutakioute, Meriem Ouali Idrissi, Najat Idrissi El Ganouni

**Affiliations:** 1 Radiology, Centre Hospitalier Universitaire Mohammed VI, Marrakech, MAR

**Keywords:** hemorrhage, dic, envenomations, viper, stroke

## Abstract

In Morocco, envenomations caused by viper bites and scorpion stings are frequent and associated with high lethality. It occurs mainly during the summer season with a peak between June and August. It is a medical emergency requiring intensive care. It is a life-threatening disease mainly due to the systemic hemorrhagic syndrome. Here, we present the clinical course of a patient in his 50s who suffered a snakebite and describe the neurological sequelae. The patient was admitted to the emergency room in a state of unconsciousness with gangrene on the right foot. An emergency brain scan showed meningeal hemorrhage and intracerebral hematomas. The biological assessment revealed disseminated intravascular coagulation. The evolution of the clinical course was rapidly unfavorable and the patient died within 24 hours of admission.

## Introduction

In Morocco, envenomation accidents remain a major public health problem. Snakebite stings and envenomations remain underestimated due to their under-reporting compared to scorpion bites and envenomations. In 2001, a national strategy was put in place by the Moroccan Poison Control Centre for the management of scorpion stings, which reduced the mortality rate by more than 80% (from 1.54% to 0.22%) [1]. However, this policy did not include snakebites, which have a mortality rate of 7.2% [2]. Hemorrhagic syndrome, necrosis, and compartment syndrome are the main local signs. The hemorrhagic syndrome usually defines the severity of these envenomations and the prognosis. Here, we report the fatal case of a 56-year-old patient.

## Case presentation

**Figure 1:**
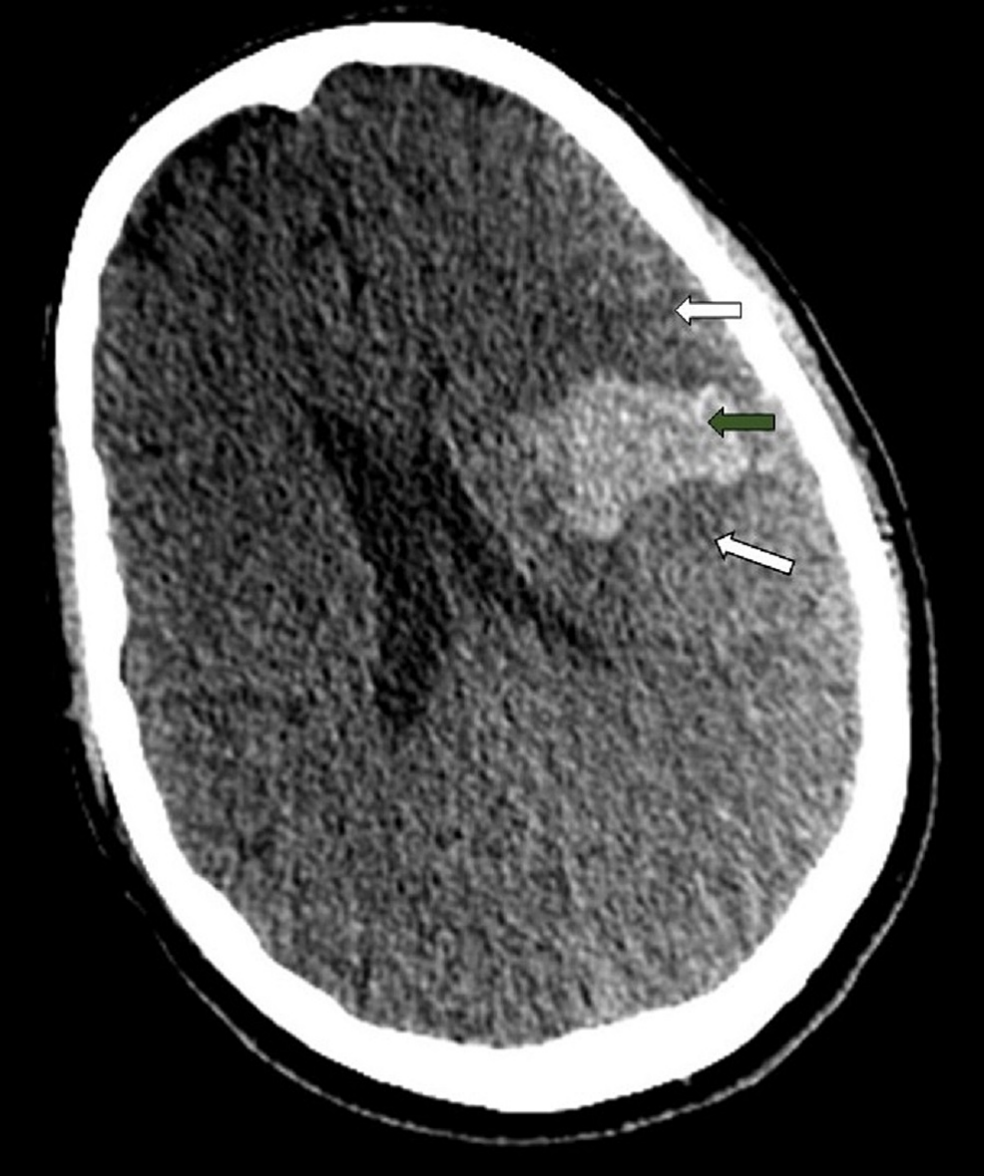
Cerebral CT, axial slice, without injection of contrast. Left deep parietal hematoma (green arrow), with perilesional edema (white arrow) and right falcique engagement. CT: computed tomography

**Figure 2:**
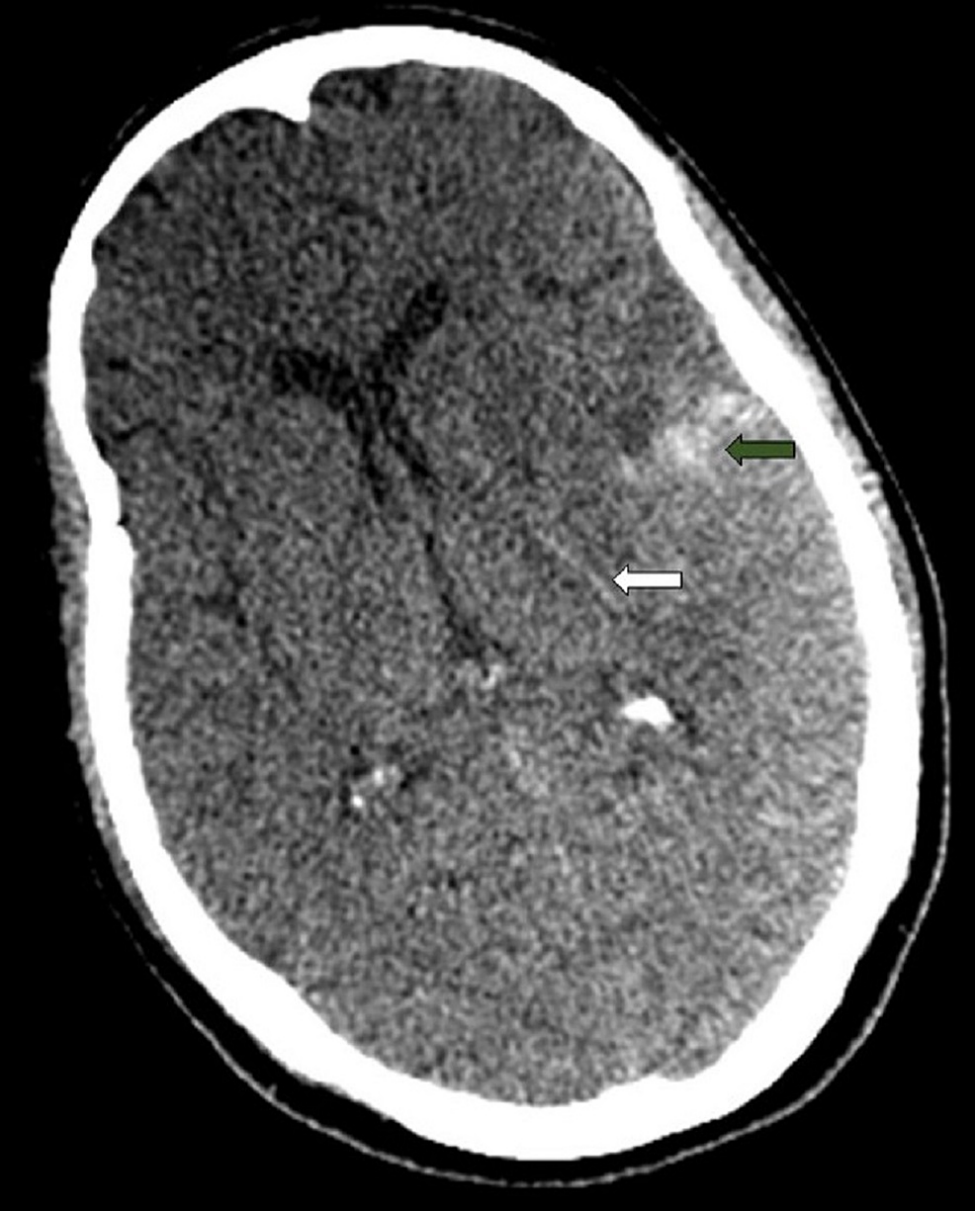
Cerebral CT, axial section, without injection of contrast. Subarachnoid meningeal hemorrhage (white arrow) and intraparenchymal hematoma (green arrow). CT: computed tomography

A 56-year-old patient with no known history was admitted to the emergency department in a state of unconsciousness for a snake bite seven days ago. The patient was intubated and ventilated. On presentation, his Glasgow Coma Scale score was 8/15, and he had right hemiplegia and right anisocoria. His arterial pressure was 10/60 cmHg. On physical examination, gangrene on the right foot was noted. The biological workup showed a hemoglobin level of 17 g/dL, thrombocytosis of 516,000 µL, and a prothrombin level of 85%. The rest of the workup showed a C-reactive protein level of 111.9 mg/L. A brain scan without injection of contrast was performed urgently and revealed a deep parietal hematoma with neighboring edema (Figure 1), associated with a meningeal hemorrhage (Figure 2) and the onset of falcique engagement. The diagnosis of a hemorrhagic stroke of viperine origin was made. The patient was immediately given an intravenous infusion of FAV-Afrique® (Sanofi-Pasteur, France) antivenom at a dose of four ampoules, along with crystalloid volume expansion, intravenous antibiotic therapy, and local care of the bite site. The patient died 24 hours after admission in a deep coma with an absence of all trunk reflexes.

## Discussion

Viperine envenomation is a deadly disease and many Moroccans remain at risk. Due to reporting standards, many cases are not officially documented [3-5]. Bites occur mainly in summers with a peak in August, as in our patient’s case. A previous study reported an envenomation rate of 80% in cases of viperine syndrome, which is considerably higher than the 30-50% envenomation rate reported in the literature [6]. The regions of Souss Massa Daraa, Guelmim Es-Smara, and Marrakech Tensift El Haouz are particularly at risk with high lethality rates. This can be explained by the geographical distribution of the Heurping viper and the Atlas viper [2-4]. Clinically, there are local signs with inflammation (constant), bite marks (inconsistent), hemorrhagic signs, compartment syndrome, and necrosis. In addition, there are general signs of hemorrhagic syndrome and shock which may be vagal, anaphylactic, or cardiogenic [7]. Neurological damage is usually the result of hemostasis disorders, the mechanism of which is complex and is explained by direct neurotoxicity by alpha, beta, and kappa neurotoxins. The proteins lectin C and disintegrin inhibit platelet aggregation and the coagulation cascade [6]. In the case of viperine envenomations, neurotoxicity is indirect and induced by tissue damage and enzyme-induced hemostasis disorders. These disorders are thought to be the cause of vascular hemorrhagic accidents, as seen in our patient. They manifest as cranial nerve palsy caused by pathognomonic ptosis, visual disturbances (diplopia), hearing, taste, swallowing, and speech, evolving to respiratory paralysis. CT scan shows a subdural or intraparenchymal hematoma with neighboring edema and meningeal hemorrhage with or without subfalcoral involvement. Few cases have been reported describing disseminated intravascular coagulation secondary to Cerastescerastes envenomation [8,9]. For management, the choice of antivenom is based on its efficacy against the venom of the identified or suspected snake biter (neutralizing power >20 LD50/mL), its adverse effects, availability, shelf-life, and duration of validity. The dose of antivenom required depends on the age and weight of the victim. It is based on the quantity of venom injected, which is very difficult to assess. Regarding the management of neurovascular complications, it can only be preventive in [10,11].

## Conclusions

Viperine envenomation, although rare, is a medical emergency requiring intensive care with symptomatic treatment. In some cases, it is life-threatening, mainly due to the systemic hemorrhagic syndrome. In the case of any suspected envenomation with loss of consciousness, a cerebral CT scan must be performed to assess the presence and extent of intracranial hemorrhage of viperine origin.
